# Microvascular decompression for trigeminal neuralgia in the elderly: efficacy and safety

**DOI:** 10.1007/s00415-020-10187-w

**Published:** 2020-08-30

**Authors:** Tobias Greve, Joerg-Christian Tonn, Jan-Hinnerk Mehrkens

**Affiliations:** Department of Neurosurgery, University Hospital, Ludwig Maximilian University of Munich, Munich, Germany

**Keywords:** Efficacy, Elderly, Microvascular decompression, Safety, Trigeminal neuralgia

## Abstract

**Objective:**

The safety and efficacy of surgical microvascular decompression (MVD) in elderly patients with trigeminal neuralgia (TN) is controversially discussed in the literature. A widespread reluctance to expose this cohort to major intracranial surgery persists. Our aim was to compare the efficacy and safety between older and younger patients with TN.

**Methods:**

In this cross-sectional study, 139 MVD procedures (103 patients < 70 and 36 patients ≥ 70) were included. Surgical fitness was assessed by the American Society of Anesthesiology (ASA) grade. The pain-free interval was evaluated using Kaplan–Meier analysis only in patients with a recent follow-up visit. Independent risk factors for recurrence in patients with a minimum 12-month follow-up were determined.

**Results:**

Patients ≥ 70 showed a significantly higher number of comorbidities. Pain intensity, affection of trigeminal branches and symptom duration was similar between groups. No significant difference in treatment associated complications and permanent neurological deficits was shown. There was no treatment-related mortality. A tendency towards a lower recurrence rate in patients < 70 did not reach statistical significance (17.6% vs. 28.6%, *P* = 0.274). Pain-free interval was not different between both cohorts (78.7 vs. 73.5 months, *P* = 0.391).

**Conclusion:**

Despite a higher prevalence of comorbidities in elderly patients, complication rates and neurological deficits after MVD were comparable to younger patients. Rates of immediate and long-term pain relief compared favorably to previous studies and were similar between elderly and younger patients. These data endorse MVD as a safe and effective first-line surgical procedure for elderly patients with TN and neurovascular conflict on MRI.

## Introduction

Classical trigeminal neuralgia (TN) is a chronic pain disorder manifesting with unilateral paroxysmal stabbing pain involving one or more divisions of the trigeminal nerve. It is the most prevalent facial pain syndrome and pain onset is usually between the ages of 40 and 60. Pain severity can hinder activities of daily living and impairs quality of life [26].

Anticonvulsant medication is the first-line therapy and can reduce TN pain intensity in 75% of patients [10]. However, the efficacy of conservative treatment generally decreases over time and TN is frequently resistant to multidrug treatment regimens. Also, these medications commonly induce side effects that lead to discontinuation of the medical therapy [39].

Around 75% of TN cases are associated with trigeminal nerve compression by a branch of the superior cerebellar artery or other blood vessels [2, 8, 12]. In cases where such a neurovascular conflict is present and where other underlying etiologic conditions such as demyelinating autoimmune diseases are ruled out, microvascular decompression of the trigeminal nerve (MVD) is the primary surgical treatment option since it is the only causal treatment for TN and offers a high rate of immediate and long-term pain relief [14, 15].

Although MVD is widely offered to younger patients, neurosurgeons tend to be reluctant to offer MVD to elderly patients, primarily because of concerns regarding complications of general anesthesia and posterior fossa surgery [3, 19, 21, 31].

However, the incidence of TN increases with age, with 4.1 per 100,000 per year in the general population [9, 20] and 20 per 100,000 per year in patients above 65 years [1]. Antiepileptic drugs used to treat TN induce side effects more frequently in elderly patients [39] and these patients are generally more sensitive to disturbances of the central nervous system, which can result in gait disturbances and ataxia and an increased tendency to fall [34]. In addition to possible comorbidities, there are physiological changes during aging, which render the serum concentration of antiepileptic drugs unpredictable [29]. One study found 25% of patients taking more than one drug were possible candidates for drug–drug interactions in the geriatric cohort [37]. Older patients are more often offered symptomatic surgical therapies such as percutaneous balloon compression [4] or radiofrequency rhizotomy [17] which access the gasserian ganglion via the oval foramen and circumvent the need for major intracranial neurosurgery. These symptomatic surgical options are however associated to poorer long-term pain control compared to MVD [24].

A higher incidence of TN in the elderly and the above-mentioned clinical particularities in this cohort combined with an overall ageing society [7] warrant a new perspective on MVD for patients in the senium.

The purpose of this study was to analyze the neurological outcome and TN recurrence rates in patients beyond 70 years of age in comparison to younger patients to add more definitive data to the mixed reports on that topic.

## Methods

### Study design

**Fig. 1:**
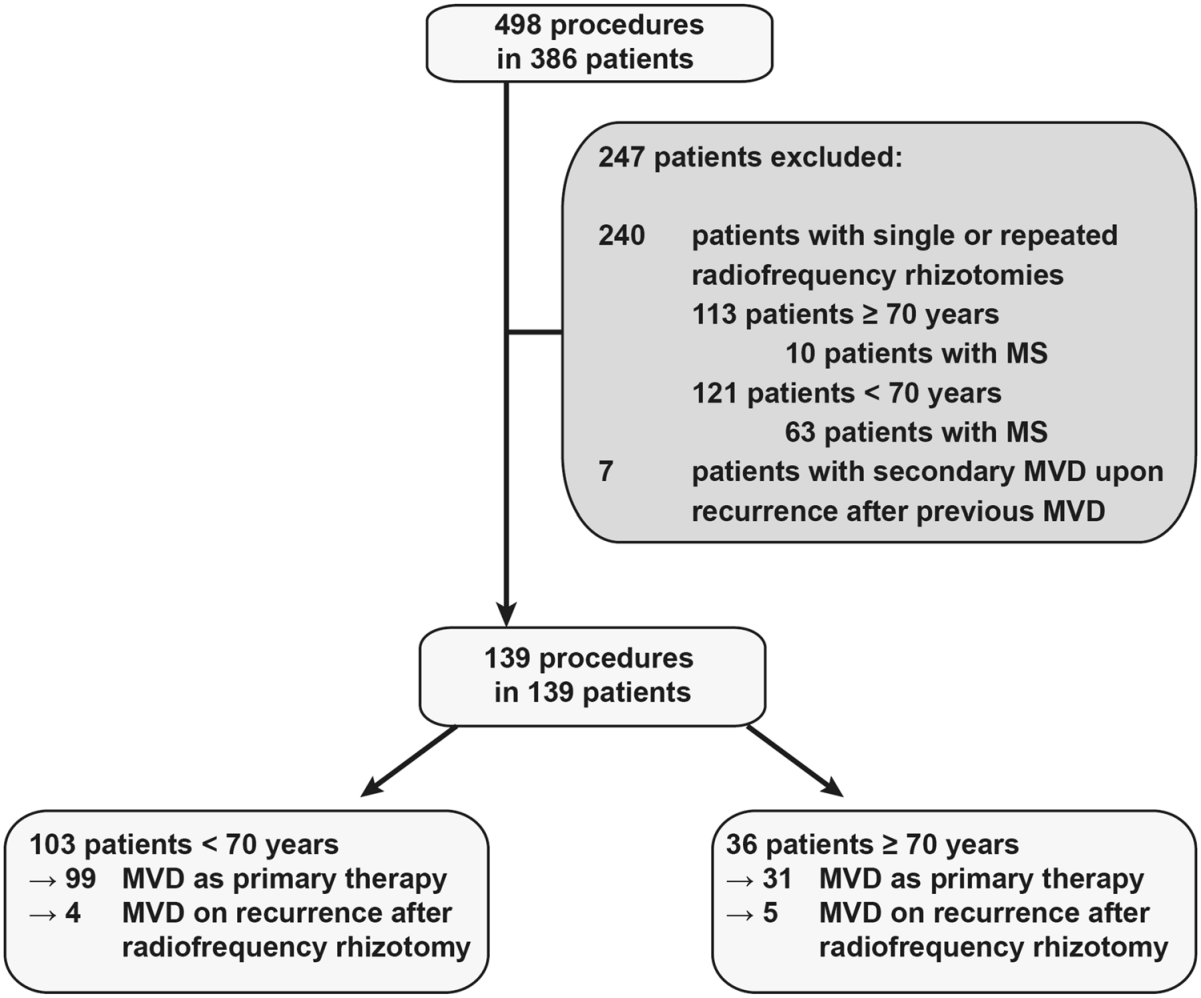
Schematic overview of patient inclusion and exclusion

In this single-center cross-sectional study, we reviewed the medical records of 485 consecutive patients undergoing surgical procedures to treat trigeminal neuralgia between 01/2012 and 02/2020. Patients who only received radiofrequency rhizotomy and who previously received MVD were excluded. Absence of a neurovascular conflict on MRI, presence of tumors within the proximity of the trigeminal nerve and presence of demyelinating autoimmune diseases were excluded. Whether the neurovascular conflict was specific to the root entry zone or whether the nerve showed atrophy was not evaluated. Consequently, a possible change in outcome depending on these MRI parameters was not part of the study. To comply with the cross-sectional design of the study, all remaining patients were actively contacted and only those who were followed up within 4 weeks of the study end (database closure 03/2020) were included. By this means, 139 MVD procedures (equaling 139 patients) were included (Fig. 1). The study duration for each patient ended upon recurrence or with the most recent follow-up. The local ethics committee board approved this cross-sectional analysis (approval number 20-233). Patient consent was waived for this study. Patients younger than 70 years of age at surgery (termed thereafter “patients < 70”) and patients that were 70 years or older (termed thereafter “patients ≥ 70”) were compared using.

### Preoperative decision-making and operative technique

All patients < 70 with TN and a visible neurovascular conflict were recommended to undergo MVD when conservative management was insufficient to control pain or when significant side effects of the medication were reported. In patients < 70 who showed relevant comorbidities, who did not show a neurovascular conflict or who had a history of an inflammatory demyelinating disease, radiofrequency rhizotomy was recommended as an alternative surgical option for TN.

Patients ≥ 70 were routinely recommended radiofrequency rhizotomy but were also offered to undergo MVD when American Society of Anesthesiology (ASA) grade was 3 or less.

Microvascular decompression was performed via a retrosigmoid approach with the patient in a modified park bench position. Intraoperative monitoring was performed in all cases. Patients were usually seen in the outpatient clinic for the first follow-up examination after 3 months.

### Analysis and outcome definitions

TN disease variables included degree of pain intensity measured by the numerical rating scale (NRS) [13, 23], degree of pain control with/without medication measured by the Barrow Neurological Institute Pain Intensity (BNI) Score [30], TN medication regimen before MVD and duration of TN symptoms before MVD. The operative fitness status was assessed by the ASA grade [11] and by identifying comorbidities.

Postoperative data included length of hospital stay, presence of neurologic deficits, pain intensity before and after MVD, oral pain medication before MVD and after MVD, complications and subsequent TN procedures. Pain intensity was determined at the first follow-up and at the most recent follow-up visit or most recent follow-up telephone interview using NRS rating and BNI Score.

### Statistics

We used Fisher's exact test and Chi-square test with Yates correction to compare distribution of categorical variables among groups. Continuous variables were tested for normal distribution using the Shapiro–Wilk test and no continuous variable was found to be normally distributed. Consequently, we employed Mann–Whitney *U* test to compare continuous variables.

Long-term pain intensity and outcome were only performed in patients who either suffered a recurrence or who had a follow-up period of at least 12 months. Long-term outcome was evaluated by Kaplan–Meier analysis with log-rank testing to compare the pain-free interval between groups. Binary logistic regression analysis was employed to find factors associated with recurrence. Statistical significance was set at *P* < 0.05. All statistical analyses were performed using SPSS version 25 (IBM).

## Results

### Demographics and characterization of trigeminal neuralgia

Of 139 patients with TN who received an MVD, 103 (74.1%) were younger than 70 years and 36 (25.9%) were 70 years or older. The age difference was statistically different as per definition of both patient cohorts (*P* < 0.001). Absolute numbers of risk factors for TN like additive headache syndromes, chronic sinusitis or previous sinus surgery were low and similar in both groups. There was a significantly higher proportion of hypercholesterolemia, arterial hypertension, carotid stenosis, and history of cancer in patients ≥ 70. Other comorbidities were not different between groups (Table 1). As a composite score of preoperative comorbidities, the above-mentioned differences reflected in the ASA status. Patients ≥ 70 had a higher frequency of an ASA status of 3 (*P* = 0.034). No patients with an ASA status of 4 or 5 underwent MVD.

**Table 1:** Patient demographics and comorbidities

Group	< 70 years	≥ 70 years	Overall	*P*
*N*	103	36	139	
Age (years)	57.6 [46.8–65.2]	73.4 [71.9–75.3]	63.4 [51.8–71.1]	** < 0.001**
Sex (females)	51 (49.5%)	17 (47.2%)	68 (48.9%)	0.813
ASA status				**0.034**
1	6 (5.8%)	–	6 (4.3%)	
2	82 (79.6%)	26 (72.2%)	108 (77.7%)	
3	15 (14.6%)	10 (27.8%)	25 (18%)	
Comorbidities				
Headache syndrome	7 (6.8%)	2 (5.6%)	9 (6.5%)	0.795
Surgery on sinuses	6 (5.8%)	–	6 (4.3%)	0.139
Fibromyalgia	4 (3.9%)	–	4 (2.9%)	0.230
Chronic sinusitis	1 (1.0%)	–	1 (0.7%)	0.553
Hypercholesterolemia	2 (1.9%)	7 (19.4%)	9 (6.5%)	**0.001**
Arterial hypertension	32 (31.1%)	18 (50.0%)	50 (36.0%)	**0.042**
Obstructive sleep apnea	5 (4.9%)	1 (2.8%)	6 (4.3%)	0.690
Anticoagulation	2 (1.9%)	2 (5.6%)	4 (2.9%)	0.572
Carotid stenosis	2 (1.9%)	6 (16.7%)	8 (5.8%)	**0.004**
TIA in the past	3 (2.9%)	1 (2.8%)	4 (2.9%)	0.967
Cardiac stents, aspirin	5 (4.9%)	7 (19.4%)	12 (8.6%)	**0.013**
Diabetes mellitus type 2	4 (3.9%)	2 (5.6%)	6 (4.3%)	0.671
Nicotine abuse	11 (10.7%)	–	11 (7.9%)	0.066
Von Willebrand disease	1 (1.0%)	–	1 (0.7%)	1.000
GERD	2 (1.9%)		2 (1.4%)	0.613
Hypothyroidism	10 (9.7%)	8 (22.2%)	18 (12.9%)	0.080
Depression	4 (3.9%)	0 (0%)	4 (2.9%)	0.572
History of cancer	1 (1%)	6 (16.7%)	7 (5%)	**0.001**

Bold text indicates a statistically significant difference

Frequencies are presented as *n* (%). Age is presented as median and interquartile range

*ASA* American Society of Anesthesiologist grading system of operative fitness, *TIA* transient ischemic attack, *GERD* gastroesophageal reflux disease

Distribution of affected branches of the trigeminal nerve was similar. Median pain intensity before MVD was 7 on the NRS in both groups. The BNI Score was 4 and 5, in 50% of patients respectively (no group difference, *P* = 0.845) (Table 2).

**Table 2:** Disease characteristics and treatment

Group	< 70 years	≥ 70 years	Overall	*P*
*N*	103	36	139	
Trigeminal branch affected				0.525
II	26 (25.2%)	7 (19.4%)	33 (23.7%)	
III	14 (13.6%)	8 (22.2%)	22 (15.8%)	
I + II	7 (6.8%)	4 (11.1%)	11 (7.9%)	
II + III	42 (40.8%)	16 (44.4%)	58 (41.7%)	
I + II + III	14 (13.6%)	1 (2.8%)	15 (10.8%)	
Side				0.401
Left	45 (43.7%)	14 (38.9%)	59 (42.4%)	
Right	58 (56.3%)	22 (61.1%)	80 (57.6%)	
Symptom duration before treatment (years)	4.0 [2.0–8.0]	4.5 [2.4–9.3]	4.0 [2.0–8.0]	0.442
Pain intensity before MVD, NRS	8 [7–8]	7 [7–8]	7 [7–8]	0.332
Pain intensity before MVD, BNI				0.845
BNI Score 4	51 (49.5%)	19 (52.8%)	70 (50.4%)	
BNI Score 5	52 (50.5%)	17 (47.2%)	69 (49.6%)	
MVD primary therapy	99 (96.1%)	31 (86.2%)	130 (93.5%)	**0.036**
MVD on recurrence after radiofrequency rhizotomy	4 (3.9%)	5 (13.8%)	9 (6.5%)	
Average length of surgery (minutes)	165 [147–199]	173 [138–205]	168 [144–201]	0.579
Average length of stay (days)	8.3 [8.1–9.9]	9.1 [8.2–10.1]	8.4 [8.1–9.9]	0.225
Immediate pain relief after MVD	99 (96.1%)	34 (94.4%)	133 (95.7%)	0.649

Bold text indicates a statistically significant difference

Frequencies are presented as *n* (%). Symptom duration, pain intensity before MVD (NRS), average length of surgery and average length of stay are presented as median and interquartile range

*MVD* microvascular decompression, *NRS* Numerical Rating Scale, *BNI Score* Barrow Neurological Institute Pain Intensity Score

Before MVD, 97.1% of patients received oral medication, the most prevalent substance being carbamazepine. Four patients were not on oral medication due to severe side effects leading to discontinuation (4 patients < 70 and 1 patient ≥ 70, *P* = 0.876).

### Treatment details and safety analysis

Most patients underwent MVD as primary surgical treatment option (96.1% in patients < 70 versus 86.2% in patients ≥ 70, *P* = 0.036), while a small percentage received MVD after failed radiofrequency rhizotomy. The median length of surgery and hospital stay was similar in both groups (Table 2).

Overall rates of transient neurological deficits were low in both groups, with transient mild facial hypoesthesia making up most of these deficits (23/139, 16.5%). Two patients < 70 required surgical revision due to deep but extradural wound infection. No other surgical revisions were required. The combined count of short-term neurological deficits and treatment related complications was not different between groups (Table 3).

**Table 3:** Complications and neurological deficits

Group	< 70 years	≥ 70 years	Overall	*P*
*N*	103	36	139	
Combined short-term complications and morbidities	22 (21.4%)	10 (27.8%)	32 (23.0%)	0.431
Transient facial numbness	16 (15.5%)	7 (19.4%)	23 (16.5%)	
Transient trochlear nerve palsy	–	1 (2.8%)	1 (0.7%)	
Transient vocal cord palsy	–	1 (2.8%)	1 (0.7%)	
Transient facial palsy after ischemia in the facial motor nucleus	–	1 (2.8%)	1 (0.7%)	
Venous sinus thrombosis with prolonged anticoagulation	1 (1%)	–	1 (0.7%)	
Cerebellar ischemia, prolonged SIADH	1 (1%)	–	1 (0.7%)	
CSF leak, lumbar drain	2 (1.9%)	–	2 (1.4%)	
Wound infection requiring surgical revision	2 (1.9%)	–	2 (1.4%)	
Surgical revision for other reasons	–	–	–	
Combined persistent deficits	8 (7.8%)	4 (11.1%)	12 (8.6%)	0.539
Persistent chronic headache	1 (1.0%)	1 (2.8%)	2 (1.4%)	
Persistent severe vertigo	1 (1.0%)	–	1 (0.7%)	
Persistent hearing impairment	2 (1.9%)	1 (2.8%)	3 (2.2%)	
Persistent facial hypesthesia	4 (3.9%)	2 (5.6%)	6 (4.3%)	
Treatment-related mortality	–	–	–	1.000

Frequencies are presented as *n* (%)

*SIADH* syndrome of inappropriate antidiuretic hormone secretion, *CSF* Cerebrospinal fluid leak

As to long-term neurological deficits, moderate postoperative hearing impairment occurred in 2 patients < 70 and 1 patient ≥ 70. Facial hypoesthesia improved over time in all patients but a small area of permanent hypoesthesia persisted in 6 patients (4 patients < 70 and 2 patients ≥ 70). Persistent chronic headache after MVD was found in one patient of each group. One patient < 70 has persistent severe vertigo, incapacitating the patient from engaging in gainful employment. The rate of persistent neurological deficits was not different for patients < 70 and patients ≥ 70 (Table 3). There was no treatment related mortality.

### Efficacy and long-term follow-up

Of all patients, 133 (95.7%) reported immediate pain relief after surgery with no significant difference between groups (*P* = 0.649) (Table 2).

The median follow-up for all patients was 25.2 months, with no significant difference between groups (Table 4).

**Table 4:** Short- and long-term follow-up

Group	< 70 years	≥ 70 years	Overall	*P*
All patients, *N*	103	36	139	
Length of follow-up (months)	24.0 [4.2–45.8]	34.4 [8.2–71.4]	25.2 [6.0–47.2]	0.066
Pain intensity first follow-up				0.579
BNI Score 1	81 (78.6%)	31 (86.1%)	112 (80.6%)	
BNI Score 2	5 (4.9%)	0 (0%)	5 (3.6%)	
BNI Score 3	15 (14.6%)	4 (11.1%)	19 (13.7%)	
BNI Score 4	2 (1.9%)	1 (2.8%)	3 (2.2%)	
BNI Score 5	–	–	–	
Patients with ≥ 12 months follow-up, *N*	74	28	102	
Length of follow-up (months)	40.2 [22.7–64.1]	58.5 [36.2–81.9]	44.6 [26.7–72.3]	**0.014**
Recurrence	13 (17.6%)	8 (28.6%)	21 (20.6%)	0.274
Time to recurrence (months)	24.0 [15.5–28.4]	13.5 [7.7–38.4]	23.4 [9.5–28.4]	0.558
Pain intensity upon recurrence				0.897
BNI Score 1	–	–	–	
BNI Score 2	1 (7.7%)	–	1 (4.8%)	
BNI Score 3	3 (23.1%)	2 (25%)	5 (23.8%)	
BNI Score 4	5 (38.5%)	5 (62.5%)	10 (47.6%)	
BNI Score 5	4 (30.8%)	1 (12.5%)	5 (23.8%)	
Management of recurrence				0.431
Surgically (RF)	7 (9.5%)	5 (17.9%)	12 (11.8%)	
Medically	6 (8.1%)	3 (10.7%)	9 (8.8%)	
Pain intensity in non-recurrent patients (last follow-up)				0.350
BNI Score 1	54 (88.5%)	18 (90%)	72 (88.9%)	
BNI Score 2	–	–	–	
BNI Score 3	6 (9.8%)	1 (5%)	7 (8.6%)	
BNI Score 4	1 (1.6%)	0 (0%)	1 (1.2%)	
BNI Score 5	0 (0%)	1 (5%)	1 (1.2%)	
Pain-free interval (months) (Kaplan–Meier analysis)	78.7 [70.9, 86.6]	73.5 [59.6, 87.4]	78.5 [71.4, 85.6]	0.391

Bold text indicates a statistically significant difference

Frequencies are presented as *n* (%). Length of follow-up and time to recurrence are presented as median and interquartile range. Pain-free interval is the result of Kaplan–Meier-Analysis and is presented as median and upper/lower boundaries of the 95% confidence interval

*BNI Score* Barrow Neurological Institute Pain Intensity Score, *RF* radiofrequency rhizotomy

At first follow-up after 3 months, 131 (94.2%) patients were pain free, with 19 of them still on a residual dose of anticonvulsant medication. Five patients (3.6%) had a good effect with occasional pain that did not reduce quality of life and did not require medication (equaling BNI Score 2). These patients were not counted as early recurrence. There were 3 patients (2 patients < 70 and 1 patient ≥ 70), that reported some pain, not adequately controlled with medications (BNI Score 4). The distribution of pain-control at first follow-up was similar between groups (Table 4).

**Fig. 2:**
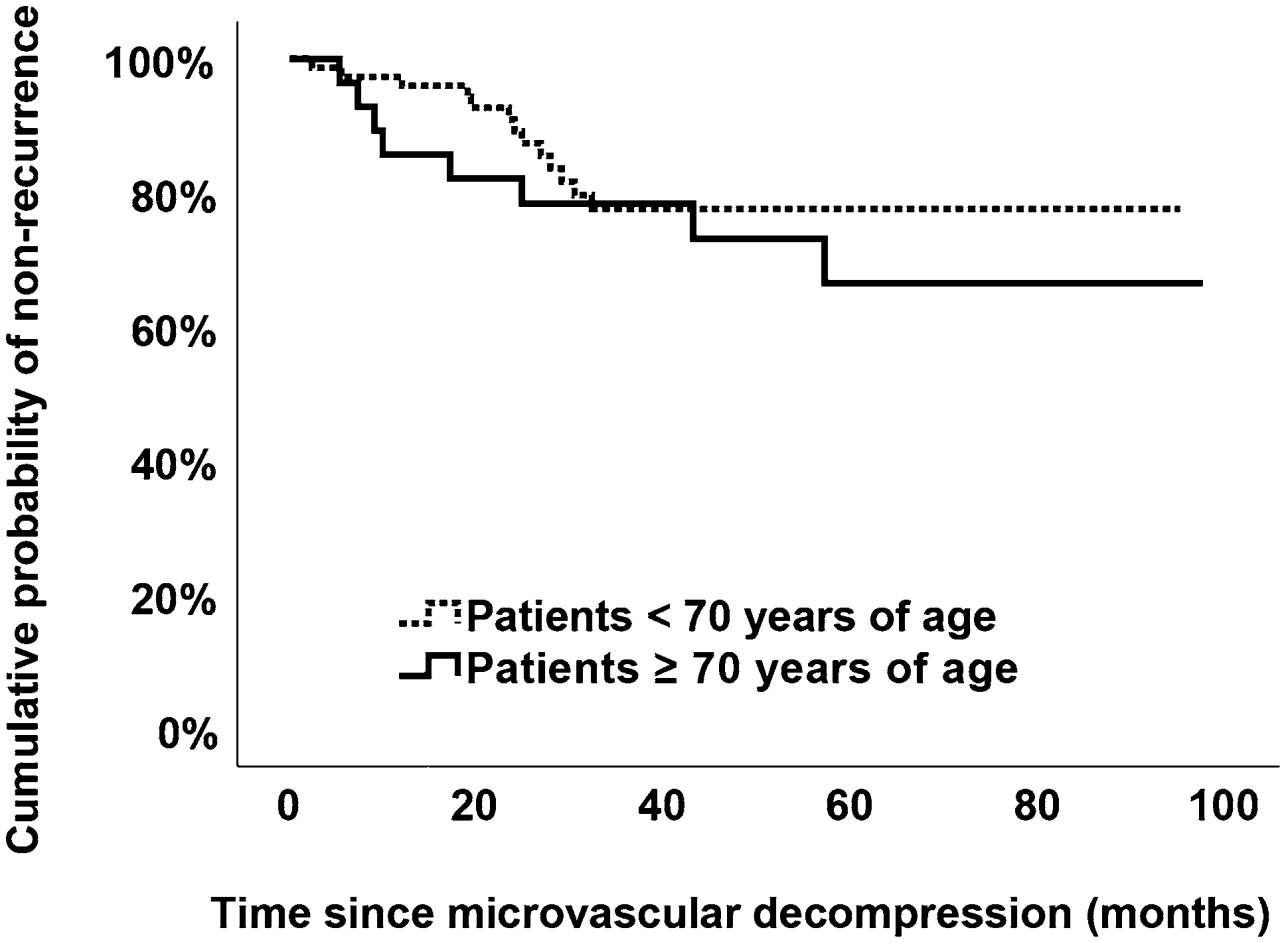
Kaplan–Meier curve of the cumulative probability of non-recurrence of trigeminal neuralgia. The cumulative probability of non-recurrence is (analogously to cumulative survival in studies with death as endpoint) the probability of non-recurrence of trigeminal neuralgia at a certain postoperative day multiplied by the probability of having no recurrence in the previous postoperative period. It did not differ between patients < 70 and patients ≥ 70 years of age (*P* = 0.391)

For long term analysis of pain control, only patients with a minimum of 12 months follow-up were analyzed. The median follow-up was 44.6 months and patients ≥ 70 had a longer follow-up (40.2 versus 58.5 months, *P* = 0.014). Within the follow-up period, there was a tendency towards higher recurrence in patients < 70 (13, 17.6%) compared to patients ≥ 70 (8, 28.6%) but the difference was not significant (*P* = 0.274). Five patients suffered from severe recurrent TN, equal to a BNI Score of 5. Pain-control and severity of recurrent TN, as measured by the BNI Score was similar between patients < 70 and ≥ 70 (*P* = 0.897). Recurrences were managed surgically by radiofrequency rhizotomy in 12 (11.8%) cases and medically in 9 (8.8%) cases (*P* = 0.431). There was a tendency towards earlier recurrence in patients ≥ 70 without statistically significant differences for both median time to recurrence (*P* = 0.558, Table 4) and Kaplan–Meier based median pain-free interval (*P* = 0.391, Fig. 2 and Table 4). Of 81 (79.4%) patients without recurrent TN at the latest follow-up visit, 79 (97.5%) were pain-free while 2 patients suffered from persistent TN inadequately controlled with medication (Table 4).

### Risk factor analysis for recurrent trigeminal neuralgia

Univariate analyses for factors associated with recurrence of TN included demographic factors, comorbidities, treatment modalities, number of medications, pre- and postoperative pain intensity as well as postoperative neurological deficits.

There was a higher recurrence rate in patients with longer symptom duration before MVD, with a median symptom duration between patients with recurrence and non-recurrence of 3 years versus 7 years (*P* = 0.006). TN not responsive to medication before MVD, equaling BNI Score 5 (*P* = 0.049), was also associated with a higher recurrence rate. Other parameters were not associated with a higher recurrence rate in univariate analysis.

For multivariate analysis, binary logistic regression was performed with the two variables proven to be associated to recurrence in univariate analysis (symptom duration and BNI Score) as well as with the variables age and ASA status. None of these variables proved as independent factor associated with a higher risk for TN recurrence after MVD **(**Table 5**)**.

**Table 5:** Multivariate analysis

Variable	*P*	Odds ratio	95% Confidence interval
Symptom duration	0.430	1.06	[0.92–1.23]
BNI Score	0.322	1.46	[0.69–3.09]
Age	0.173	0.96	[0.91–1.02]
ASA status	0.803	0.77	[0.10–5.77]

*BNI Score* Barrow Neurological Institute Pain Intensity Score, *ASA* American society of anesthesiologist grading system of operative fitness

## Discussion

In this cross-sectional study, patients with TN above and below age 70 were compared regarding efficacy and safety of MVD. Patients ≥ 70 showed a significantly higher number of comorbidities and a higher proportion of preoperative ASA 3 status. Albeit the higher age and the higher number of comorbidities, no significant difference in treatment associated complications, permanent neurological deficits and—most importantly—in number of recurrences nor in the pain-free interval was shown (Fig. 2).

In terms of demographic factors patients were comparable to other studies [16, 31]. The reported rate of immediate pain relief—usually 80–95% in the literature [32, 41], and of recurrence rates—reported between 5 and 30% in the literature [5, 6, 22, 35], were comparable to the results of the present study, where we showed 95.7% immediate pain relief and a recurrence rate of 20.6%.

The most frequently occurring neurological deficit in our study was facial hypoesthesia, with most cases resolving completely. In 6 cases (4.3%), a small patch of facial mild hypoesthesia persisted, a rate that compares favorably to the literature [5, 36].

One large study found symptom duration to be positively correlated to TN recurrence [5], a finding that was recapitulated in this series. However, it failed to remain an independent risk factor in multivariate analysis. Consistent with this finding, it was previously shown that microstructural changes in the trigeminal nerve, as determined by diffusion tensor imaging MRI, are independent of symptom duration [25].

Most studies did not report detailed comorbidities or ASA status [16, 28, 33], and one study only included elderly patients with an ASA 1 and 2 status [3]. By contrast, almost 30% of patients ≥ 70 in our study were classified as ASA 3. Albeit the clear gap in comorbidities between age groups, we report no difference in postoperative complications or permanent neurological deficits. The difference in comorbidities between groups thereby does not represent a limitation but a prerequisite to draw adequate conclusions. One large retrospective study analyzing data from 3273 patients out of a nationwide database showed that procedure-related mortality increased with age with a mortality rate of 1.2% for patients over 75 years. However, no data for preoperative comorbidities of any kind nor ASA status was included in that analysis and there was no report on outcome [31].

Another important factor presented here is the Kaplan–Meier based pain-free interval analysis which previous studies lack [16, 18, 28, 31, 33]. We were able to show that not only the number of recurrences, but also the pain-free interval is similar between patients above and below the age of 70. This is a crucial finding since a longer pain-free interval directly translates into less disability, depression and anxiety, all of which have been unequivocally linked to TN [40].

Discussing alternative surgical treatment options, a large meta-analysis with 2163 patients showed that MVD had a lower number of recurrences compared to radiofrequency rhizotomy, reducing the risk by around 66% [24]. Other groups also showed that while pain control ranges at around 80% for MVD over the course of 10 years [32, 41], there were almost 30% recurrences after 3 years for radiofrequency rhizotomy throughout all age groups [27, 38].

The superior long-term outcome of MVD over symptomatic surgical treatment options underscores the favorable risk profile in the older patient cohort and provides another strong argument for MVD as a primary surgical treatment option.

Our study stands out due to its cross-sectional design since long-term outcome was only evaluated in patients who were followed-up within the last 4 weeks. In contrast, previous studies evaluated patients during routine visits but not in a cross-sectional sense at a recent time point [18, 33].

There are two main limitations to our study. First, the small sample size of patients ≥ 70 might render subgroup analysis less robust, especially in binary logistic regression modelling. Second, a selection bias inevitably occurs since patients ≥ 70 with severe contraindications to major intracranial surgery were directed towards radiofrequency rhizotomy or stereotactic radiosurgery. This must be considered when counseling patients towards the right treatment.

Despite these limitations, this study offers convincing new aspects that substantiate the justification of MVD in elderly patients.

## Conclusion

In this study on the controversial topic of MVD in elderly patients with trigeminal neuralgia, we analyzed cohorts above and below 70 years of age with regard to efficacy and safety of MVD. We showed that MVD is equally safe and efficient despite a higher number of comorbidities in elderly patients. There was no significant difference in immediate and long-term pain relief between age groups and recurrence rates compared favorably to reports on symptomatic surgical treatments such as radiofrequency rhizotomy. The relevance and novelty of our findings lies in the cross-sectional study design, the higher proportion of relevant preoperative comorbidities in the older age group and the Kaplan–Meier based analysis of the pain-free interval. Our study endorses MVD as routine surgical procedure for TN in elderly people as long as major contraindications to intracranial surgery are ruled out.
